# Exploring the potential of standalone and tandem solar cells with Sb_2_S_3_ and Sb_2_Se_3_ absorbers: a simulation study

**DOI:** 10.1038/s41598-023-49269-w

**Published:** 2023-12-19

**Authors:** Z. Dahmardeh, M. Saadat

**Affiliations:** Department of Physics, University of Sistan and Baluchestan, Zahedan, Iran

**Keywords:** Materials science, Materials for devices, Electronic devices

## Abstract

Thin-film antimony chalcogenide binary compounds are potential candidates for efficient and low-cost photovoltaic absorbers. This study investigates the performance of Sb_2_S_3_ and Sb_2_Se_3_ as photovoltaic absorbers, aiming to optimize their efficiency. The standalone Sb_2_S_3_ and Sb_2_Se_3_ sub-cells are analyzed using SCAPS-1D simulations, and then a tandem structure with Sb_2_S_3_ as the top-cell absorber and Sb_2_Se_3_ as the bottom-cell absorber is designed, using the filtered spectrum and the current matching technique. The optimal configuration for maximum efficiency is achieved by adjusting the thickness of the absorber layer. The results show that antimony chalcogenide binary compounds have great potential as photovoltaic absorbers, enabling the development of efficient and low-cost solar cells. A remarkable conversion efficiency of 22.2% is achieved for the optimized tandem cell structure, with absorber thicknesses of 420 nm and 1020 nm for the top and bottom sub-cells respectively. This study presents a promising approach towards high-performance tandem solar cells.

## Introduction

Research is being conducted into alternative energy sources in order to identify options that are both cost-effective and environmentally friendly. As part of this research, bulk and thin film materials are being investigated as potential photovoltaic absorbers^1–5^. There has been increasing interest in the photovoltaic applications of antimony chalcogenide binary compounds, including Sb_2_S_3_, Sb_2_Se_3_, and mixed antimony chalcogenide Sb_2_(S,Se)_3_^6–10^. As well as having a high optical absorption coefficient (> 10^5^ cm^−1^ in the visible range), antimony chalcogenide binary compounds have a long carrier lifetime, adjustable band gaps (1.2 eV for Sb_2_Se_3_ and 1.7 eV for Sb_2_S_3_), decent carrier mobility, earth-abundant, long-term stability, low toxicity, moisture, and air stability^6,11–17^. Also, antimony chalcogenide thin films have been effectively prepared using both vacuum and non-vacuum processes^18–21^. Maximum efficiency of more than 10% has been achieved with antimony chalcogenide-based solar cells^22^. These characteristics make antimony chalcogenide compounds promising alternatives to existing thin-film photovoltaic technologies.

A typical single-junction solar cell uses only a portion of the solar spectrum; the rest is reflected or lost as heat (Shockley-Queisser limit). By using tandem solar cells, the Shockley-Queisser limit of single-junction solar cells can be exceeded, resulting in increased power generation under certain light conditions^8^. Tandem solar cells consist of a top cell with a high band gap that absorbs short wavelength solar radiation and a bottom cell with a low band gap that absorbs longer wavelength solar radiation. An open circuit voltage (V_OC_) for a tandem device is predicted to be the sum of the V_OC_s for each of its sub-cells. It is important to note, however, that the current density of a serial tandem cell is limited to that of the lower current density of the sub-cell, resulting in a large current loss when the current densities of the sub-cells are mismatched^23,24^. A major objective of this project is to develop the most efficient solar cell possible, which will consist of several layers with different band gaps. Having a band gap of 1.2 eV, Sb_2_Se_3_ is an excellent candidate for the bottom cell material in tandem solar cells, while Sb_2_S_3_, with a band gap of 1.7 eV, is an excellent candidate for the top cell material^8,25,26^.

Sb_2_S_3_ has been suggested as a possible top sub-cell candidate for coupling with c-Si, CIGSe, CdTe, Cu_2_ZnSnSe_4_, and Cu_2_ZnSn(S, Se)_4_ as bottom sub-cell candidates^8,27^. For example in a study conducted by Okil et al., the researchers focused on Sb_2_S_3_/Si tandem solar cells. They performed optimizations on various parameters to improve the cell's performance. As a result of these optimizations, they achieved an impressive efficiency of 23.25%^28^. Alanazi et al., conducted a simulation study introducing a 2T Organic/Sb_2_Se_3_ tandem device. The study explored inverted (p-i-n)/(p-i-n) and conventional (n-i-p)/(n-i-p) configurations. Through optimization and current matching, the inverted tandem cell achieved an impressive power conversion efficiency (PCE) of 21.52%, while the conventional tandem cell achieved a PCE of 19.14%. The initial standalone top and bottom cells exhibited efficiencies of 9.45% and 7.89% respectively^29^. The crystal structures and characteristics of Sb_2_S_3_ and Sb_2_Se_3_ are, however, similar. By utilizing these materials, tandem cells may be able to be made that are stable, cost-effective, and environmentally friendly^8,25,30^. In 2020, Zhang et al., developed a tandem solar cell utilizing antimony chalcogenide compounds. By employing Sb_2_S_3_ and Sb_2_Se_3_ as the top and bottom cells, respectively, they achieved an efficiency of 7.93%, surpassing the efficiencies of the individual top and bottom single solar cells. This study highlights the potential of antimony chalcogenides for tandem solar cell applications^31^. Cao’s study revealed a remarkable efficiency of 26.64% for Sb_2_S_3_/Sb_2_Se_3_ tandem solar cells after removing the defect state in the light-absorbing layer^25^. The recent simulation of tandem solar cells with the structure Mo/Sb_2_Se_3_/Sb_2_S_3_/CdS/i-ZnO/ZnO:Al was carried out by Bal et al. A tandem cell with 13.2% efficiency was achieved after optimization of the different parameters^32^. Dahmardeh et al., investigated how the performance of Sb_2_(S,Se)_3_/Sb_2_(S,Se)_3_ tandem solar cells changed with different amounts of selenium in the absorber layers. They found that the energy gap between the two sub-cells influenced the current flow. They obtained the best conversion efficiency of 22.19% by adjusting the Se/(Se + Se) ratio in the absorber layers^33^.

In this study, we created and analyzed single Sb_2_S_3_ and Sb_2_Se_3_ solar cells, as well as tandem Sb_2_S_3_/Sb_2_Se_3_ devices using the Solar Cell Capacitance Simulator (SCAPS) software^34^. We will first discuss the modeling and simulation results for the singular Sb_2_S_3_ and Sb_2_Se_3_ solar cell structures. In the next step, we will report the results of our simulation of tandem Sb_2_S_3_/Sb_2_Se_3_ solar cells. During this configuration, Sb_2_S_3_ acts as the absorber layer at the top of the cell and Sb_2_Se_3_ acts as the absorber layer at the bottom of the cell. Since mismatched current density is the primary source of current loss in tandem cells, it was decided to adjust the absorber thickness of the sub-cells in order to determine the “current matching” point where the sub-cells possess equal short circuit current densities. In comparison with the individual Sb_2_S_3_ and Sb_2_Se_3_ devices, the tandem cell combining these two materials demonstrated enhanced photovoltaic efficiency.

## Methodology

**Figure 1:**
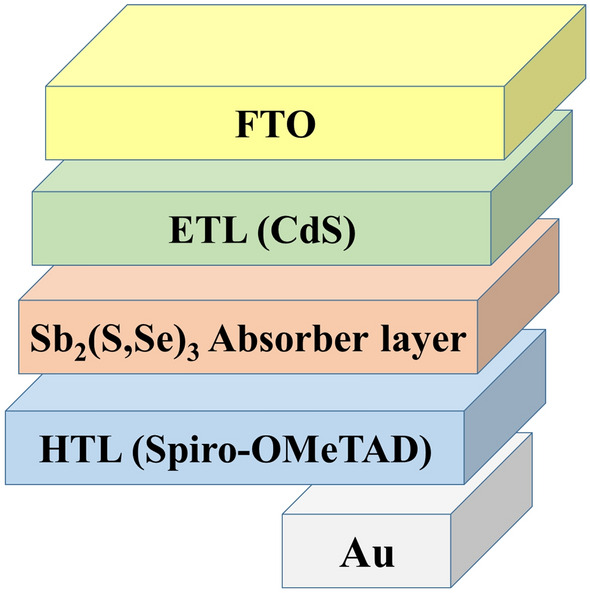
Device architecture of Sb_2_(S,Se)_3_ solar cell used in the numerical simulation.

An example of a single junction solar cell is shown in Fig. 1. As the absorber layer, this device contains Sb_2_S_3_ or Sb_2_Se_3_, CdS as the electron transport layer (ETL), and SpiroOMeTAD as the hole transport layer (HTL), as well as Au and FTO acting as back and front contacts, respectively. Simulations of single junction devices were conducted using the SCAPS software. The program is based on numerical solutions of the Poisson equation, current density equations for electrons and holes, and continuity equations for holes and electrons with appropriate boundary conditions at interfaces and contacts^13,35^.

In the first step, a model based on experimental data^22^ was used to design independent single junction sub-cells comprised of SnO_2_:F/ETL (CdS)/absorber/HTL (SpiroOMeTAD)/Au. Based on the previous research work reported in the literature, we have selected the material parameters and presented them in Table 1S^13,33,36^. We have used different dielectric permittivity and electron and hole mobility for the Sb_2_S_3_/Sb_2_Se_3_ absorber layer^25^. A neutral defect with a density of 1.4 × 10^12^ cm^−2^ was assumed at both the ETL/absorber and HTL/absorber interfaces. We have also taken into account the bulk defects reported for record Sb_2_(S, Se)_3_ solar cells in the absorber layers^22^. In addition, we have assumed an internal defect near the midgap states for HTL, ETL, and FTO layers. The defect state parameters for the absorber, HTL, ETL, and FTO layers are shown in Table 2S. We have obtained all the other input parameters for the simulation of solar cells from the literature^36^ or logically assumed them to prevent misleading or unrealistic results. The spectral conditions for all simulations are 1.5 AM and the operating temperature is 300 K. Table 3S shows a comparison of the experimental and simulated solar cells data. The good consistency between the simulated results and the experimental data confirms the validity of our model.

There are two types of absorber layers in the top and bottom sub-cells, with Sb_2_S_3_ on the top and Sb_2_Se_3_ on the bottom. A relationship was established between the performance of the sub-cells and the thickness of the absorber layer. We used the AM 1.5 G spectrum and a temperature of 300 K for standalone simulations of top and bottom sub-cells.

An absorber layer’s band gap determines the number of photons that can be absorbed by a single junction solar cell. It is possible to enhance the performance of photovoltaic systems by using tandem structures. In tandem cells containing Sb_2_S_3_/Sb_2_Se_3_, the top cell contains an absorber layer with a band gap of 1.7 eV and the bottom cell contains an absorber layer with a band gap of 1.2 eV. After evaluating the performance of Sb_2_S_3_-based top and Sb_2_Se_3_-based bottom cells, the Sb_2_S_3_/Sb_2_Se_3_ tandem structure was analyzed.

We can simulate a tandem architecture in SCAPS by connecting the top and bottom cells using the current matching technique^23^. The Sb_2_S_3_ top cell is illuminated by the standard global AM 1.5 G spectrum; however, the Sb_2_Se_3_ bottom cell is illuminated by the residual solar spectrum after partial absorption across the top cell. Using this equation^24^, we can calculate the spectrum passing through the top sub-cell with varying Sb_2_S_3_ thicknesses to the bottom sub-cell:

1 $$T\left( \lambda \right) = T_{0} \left( \lambda \right){\text{exp}}\left[ {\mathop \sum \limits_{k = 1}^{n} - \left( {\alpha_{k} \left( \lambda \right)t_{k} } \right)} \right]$$

**Figure 2:**
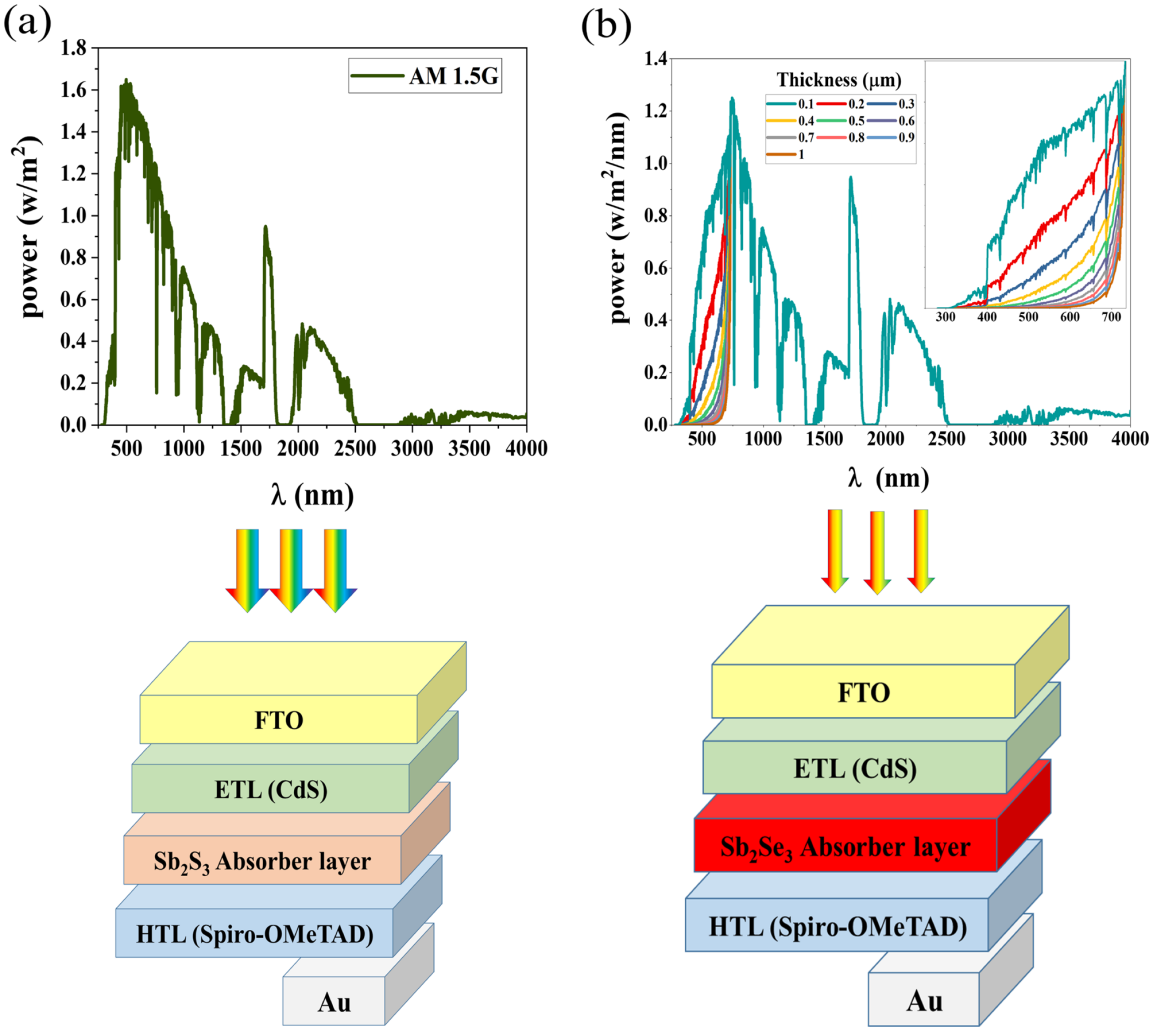
Schematic diagram of tandem cell with (**a**) AM1.5 spectrum at the top sub-cell and (**b**) filtered spectrum transmitted by top sub-cell with different absorber layer thickness at bottom sub-cell.

This is the incident standard global AM 1.5 G spectrum, the layer number, represents the number of layers of the top sub-cell, and the thickness of each layer represents T0(λ), k, n and t respectively. Also, α(λ) is the absorption coefficient of each material and given by^24^:

2 $$ \alpha \left( E \right) = A_{\alpha } \sqrt {hv - E_{g} }  $$

where Aα, Eg, h, and ν are pre-factor, the energy gap of the material (eV), the Planck's constant (eV.sec), and the spectrum frequency respectively. The Fig. 2 illustrates the schematic of a Sb_2_S_3_/Sb_2_Se_3_ tandem device with an incident AM 1.5 G spectrum to the Sb_2_S_3_ top sub-cell and a filtered spectrum to the Sb_2_Se_3_ bottom sub-cell. Each sub-cell has an absorber layer that absorbs photons with an energy greater than the band gap. The thickness of top and bottom sub-cells was altered in order to achieve equal current density across both. The monolithic tandem solar cell must meet this requirement.

## Results and discussions

### Simulation of Sb_2_S_3_ top cell

**Figure 3:**
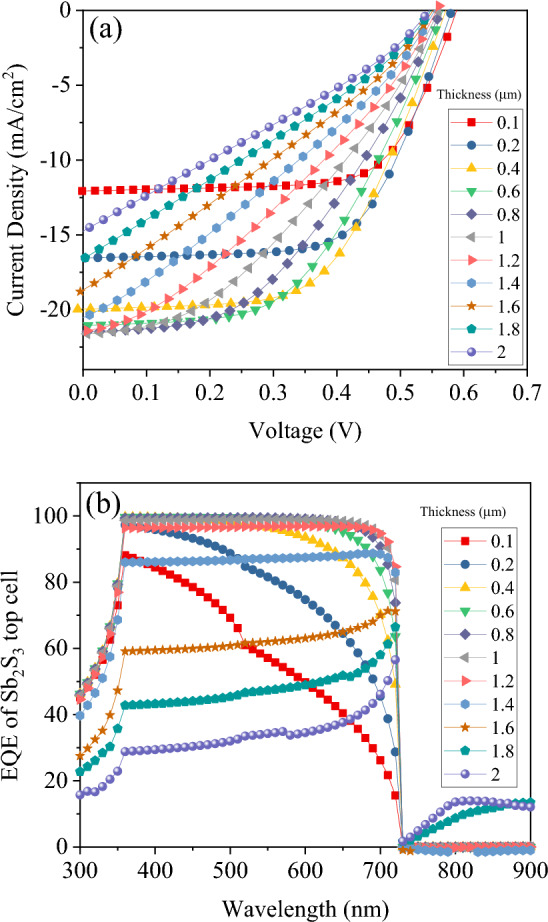
(**a**) J–V curve and (**b**) EQE of Sb_2_S_3_ based top cell with absorber thickness in a standalone configuration.

**Figure 4:**
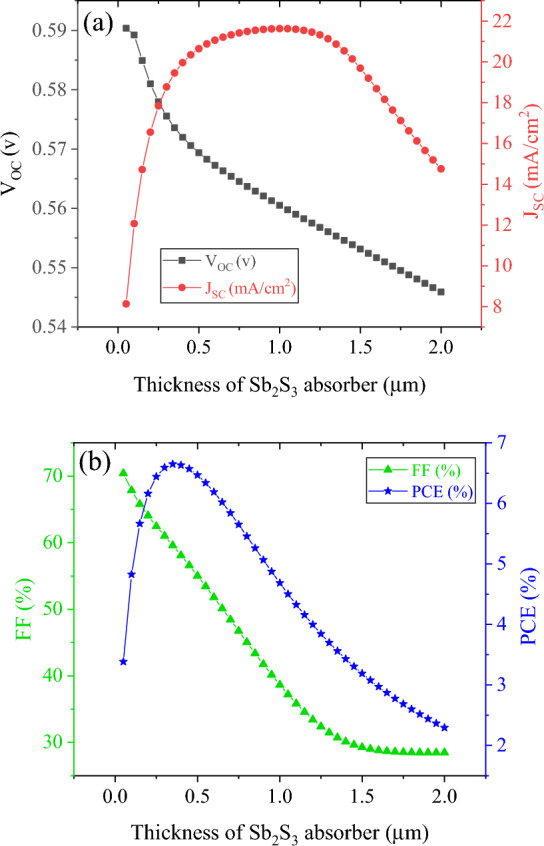
Photovoltaic characteristics of Sb_2_S_3_ based top cell with absorber thickness: (**a**) V_OC_ and J_SC_ and (**b**): FF and PCE.

**Figure 5:**
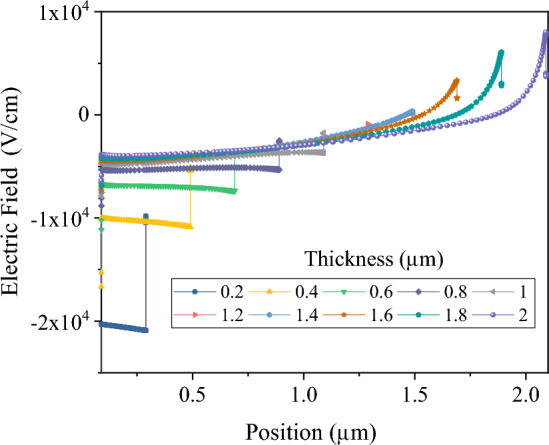
Electric field strength across the top cell Sb_2_S_3_ absorber layer with absorber thickness.

This section focuses on simulating the Sb_2_S_3_ top sub-cell. Simulations of top sub-cells were conducted using the standard AM 1.5 G spectrum. A study was conducted to examine the effect of the thickness of the absorber layer on the Sb_2_S_3_ sub-cell. The thickness of the absorber layer was varied between 50 nm and 2 µm. Figures 3 and 4 present the J–V characteristics, external quantum efficiency (EQE), and photovoltaic parameters, respectively, of Sb_2_S_3_ solar cells with varying absorber layer thicknesses. By increasing the absorbance layer thickness to 650 nm, J_SC_ increased to 21.2 mA cm^−2^ as a result of increased absorption and generation rates. Further increases in thickness beyond 650 nm, however, resulted in saturation of absorption and little change in J_SC_ values. There is an increase in recombination in the thicker Sb_2_S_3_ absorber as a result of the addition of a 1.25 µm thick Sb_2_S_3_ layer^23^. According to external quantum efficiency spectra, photon harvesting was improved up to 1–1.2 µm, after which it began to decline for wavelengths less than 730 nm. As a result of this decrease in response, J_SC_ values decreased. As the thickness of the Sb_2_S_3_ absorber increased from 50 nm to 2 µm, V_OC_ reduced slightly from 590 to 545 mV and fill factor (FF) from 70.42 to 28.47%. The reduced electric field across the thick Sb_2_S_3_ layer is the cause of the decreased V_OC_ and FF in the device (Fig. 5). The lower electric field reduces the probability of charge carrier separation, thus reducing the V_OC_ and FF^23,37^. An increase in J_SC_ increases power conversion efficiency (PCE) for thicknesses less than 350 nm. In contrast, when it comes to thicker films, it is the reduction of V_OC_s and FF that becomes the determining factor. Accordingly, PCE was initially increased, followed by a decrease as thickness increased, with a maximum PCE value of 6.65% at 350 nm thick Sb_2_S_3_.

### Simulation of Sb_2_Se_3_ bottom cell

**Figure 6:**
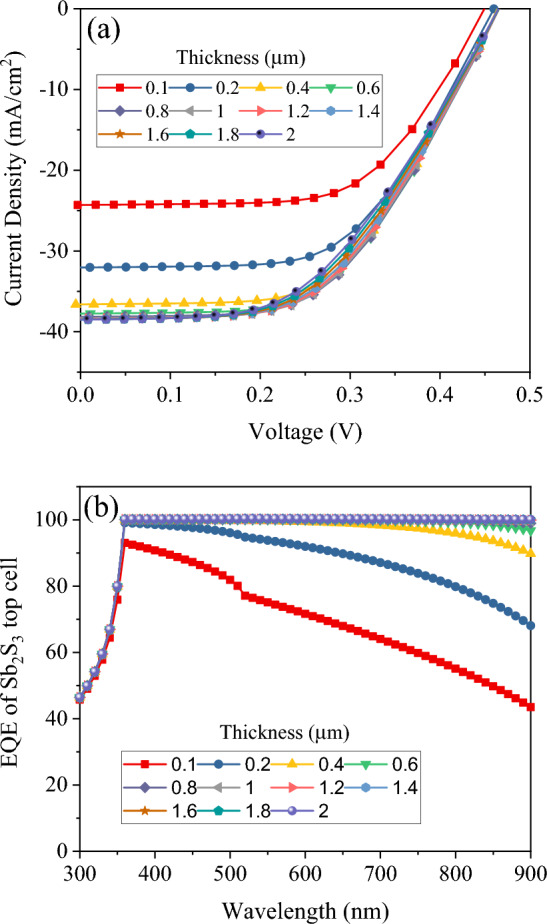
(**a**) J–V curve and (**b**) EQE of Sb_2_Se_3_ based bottom cell with absorber thickness in a standalone configuration.

**Figure 7:**
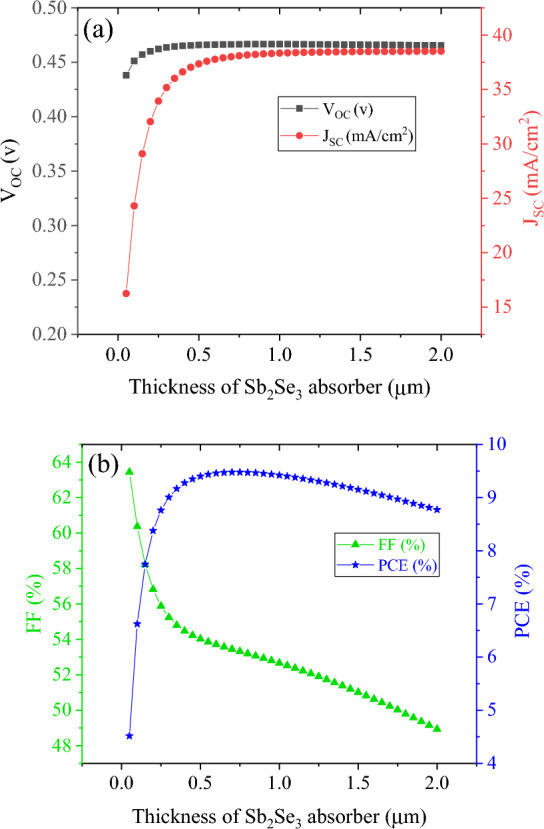
Photovoltaic characteristics of Sb_2_Se_3_ based bottom cell with absorber thickness: (**a**) V_OC_ and J_SC_ and (**b**): FF and PCE.

In this section, the study of simulating the Sb_2_Se_3_ bottom sub-cell is performed. For standalone simulations of bottom sub-cell, the standard AM 1.5 G spectrum was employed. J–V characteristics of the Sb_2_Se_3_ bottom sub-cell for different values of absorbing layer thickness are shown in Fig. 6a. Extracted from the different J–V characteristics in Fig. 6, the photovoltaic parameters of Sb_2_S_3_ solar cells with different absorber thickness is illustrated in Fig. 7. We discovered that Sb_2_Se_3_ solar cells have a relatively lower open circuit voltage than Sb_2_S_3_ absorber layer-based solar cells, as shown in Fig. 7a, because Sb_2_Se_3_ has a smaller band gap than Sb_2_S_3_ absorber layer^38,39^. A slight increase in the V_OC_ of Sb_2_Se_3_ solar cells is observed as thickness of absorber grows, reaching its maximum value. This is mostly due to the fact that a thicker Sb_2_Se_3_ absorber layer will absorb more photons, contributing to the production of electron–hole pairs^40^. Figure 7a shows that increasing the absorbance layer thickness up to 700 nm resulted in an increase in J_SC_ to 38.0 mA cm^−2^ due to enhanced absorption and generation rates. However, further increases in thickness beyond 700 nm led to saturation of absorption and little change in J_SC_ values. The external quantum efficiency spectra of Sb_2_S_3_ sub-ells with various thicknesses are depicted in Fig. 6b. The absorber layer’s photon harvesting improved with an increase in thickness of up to 0.8 µm. However, as thickness rises above this point, the optical absorption virtually reaches saturation. This also verifies the variation in the J_SC_ as mentioned earlier. In Fig. 7b, it is visible that the FF is greatly dropped from 63.4% for 50 nm to 48.9% for 2.0 µm. As the thickness of these single junction solar cells increases, so does their efficiency, peaking at 0.5–0.7 µm. After that, efficiency starts to drop off as thickness continues to increase.‏

### Simulation of Sb_2_S_3_/Sb_2_Se_3_ tandem solar cell

**Figure 8:**
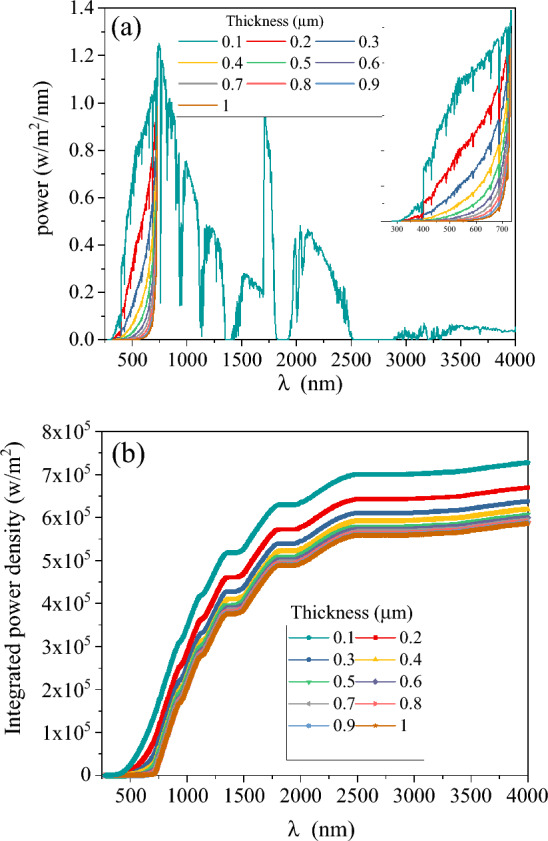
(**a**) Filtered spectrum and (**b**) integrated power density transmitted by top cell with different absorber layer thickness.

In this part, the performance of Sb_2_S_3_/Sb_2_Se_3_ tandem solar cells is explored utilizing a filtered spectrum (described in the “Methodology” section) combined with a current matching process. The filtered spectrum and integrated power are shown in Fig. 8 for different thicknesses of the absorber layer in the top cell. The filtered spectrum clearly illustrates that with increasing Sb_2_S_3_ thickness, power passed to the bottom cell decreases, especially at wavelengths below 730 nm. Furthermore, Fig. 8b shows that the integrated power transmitted by the top cell decreases as the thickness of the absorber layer decreases.

Having equal currents in both sub-cells is crucial for monolithic tandem solar cells^23,24^. Thus, both the upper and lower sub-cell thicknesses must be adjusted at the same time in order to achieve current matching. For each sub-cell, the thickness of the top cell was altered in 100 nm steps while the thickness of the bottom cell was changed until an identical J_SC_ was obtained. In the top cell, AM 15 G spectrum is used to illuminate the cell, and in the bottom cell, a filtered spectrum is used to illuminate the cell. Therefore, the J_SC_ for the lower sub-cell is altered as a result of the thickness of the absorb layer in both cells, while for the upper cell the J_SC_ is altered as a result of the thickness of the absorb layer in only one cell.

**Figure 9:**
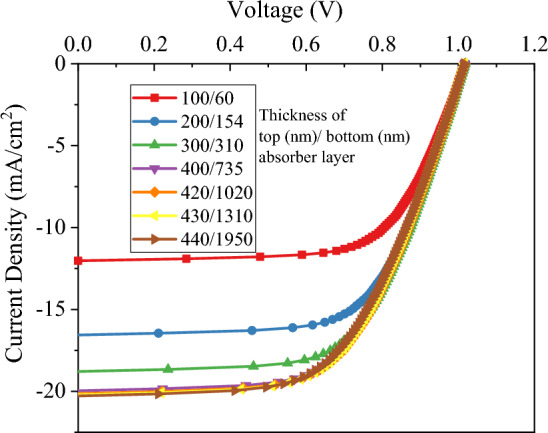
J–V curve of Sb_2_S_3_/Sb_2_Se_3_ tandem with thicknesses corresponding to first seven current matching points.

**Figure 10:**
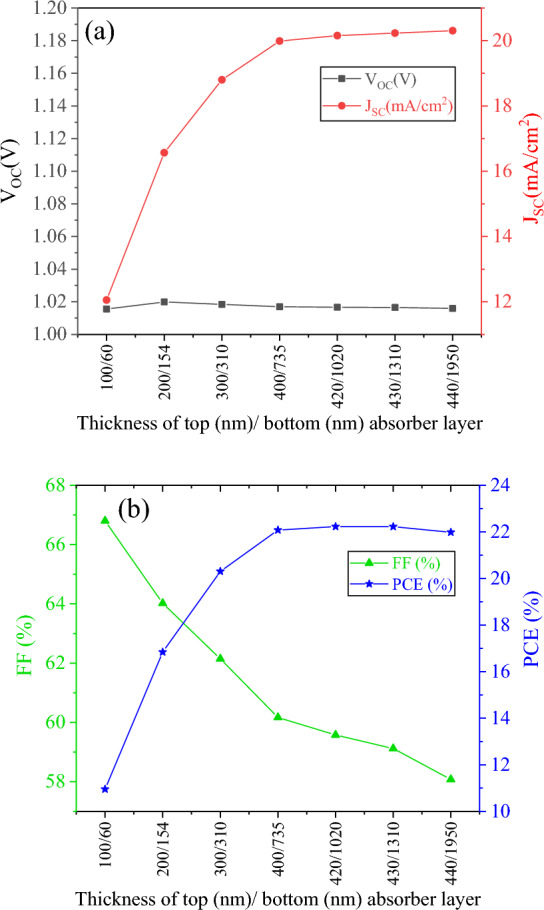
Photovoltaic characteristics of Sb_2_S_3_/Sb_2_Se_3_ tandem solar cell with thicknesses corresponding to the first seven current matching points: (**a**) V_OC_ and J_SC_ and (**b**): FF and PCE.

**Figure 11:**
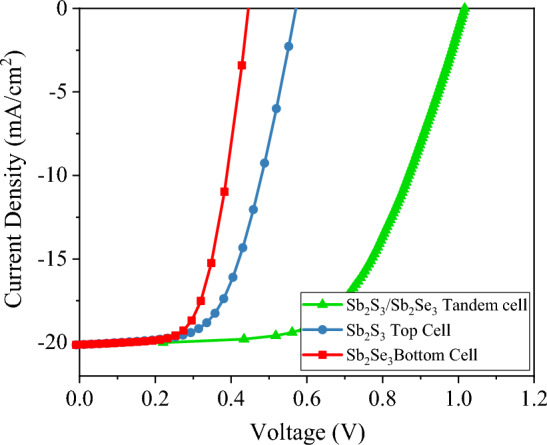
Illuminated J–V curves of standalone Sb_2_S_3_ top and Sb_2_Se_3_ bottom sub-cells at optimized thickness (420 nm/1020 nm), bottom cell under filtered spectrum by top cell, and Sb_2_S_3_/Sb_2_Se_3_ tandem solar cell.

J-V curves are presented in Fig. 9 for different thicknesses of Sb_2_S_3_/Sb_2_Se_3_ tandem solar cells. At different current matching points, Fig. 10 illustrates the fluctuation of photovoltaic characteristics of tandem solar cells. Compared to the single device, the tandem device exhibits improved values of J_SC_ at higher current matching points. With a 430 nm (top absorber)/1310 nm (bottom absorber) configuration, the highest J_SC_ value was achieved. Current matching requires thicker bottom absorbers for thicker top absorbers; for example, 450 nm thick top absorbers require 6 µm thick bottom absorbers. In order to minimize material consumption, keeping the top layer within 400–430 nm is more efficient. A comparison of V_OC_ of tandem solar cells for different current matching points shows that V_OC_ to FF ratios decrease at higher current matching points. As shown in Fig. 10b, the PCE of the tandem cell under matching conditions can be determined. As cell thickness increases, efficiency increases until it reaches a plateau at or beyond 400 nm for the upper sub-cell. 420 nm and 1020 nm are the optimal thicknesses for the top and bottom sub-cells of a tandem device (Fig. 10b). The efficiency of the system becomes saturated when the value is exceeded. Figure 2 illustrates the structure of this device, illuminated by AM 1.5 G incident spectrum on the top sub-cell of Sb_2_S_3_ and filtered AM 1.5 G on the bottom sub-cell of Sb_2_Se_3_. Figure 11 shows the J–V curve of each sub-cell along with their combined tandem solar cell counterpart.

**Figure 12:**
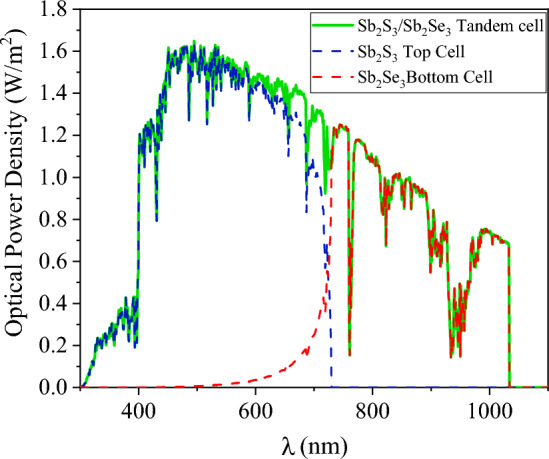
The absorbed spectrum of Sb_2_S_3_, Sb_2_Se_3_ sub-cells, and Sb_2_S_3_/Sb_2_Se_3_ tandem cell.

**Figure 13:**
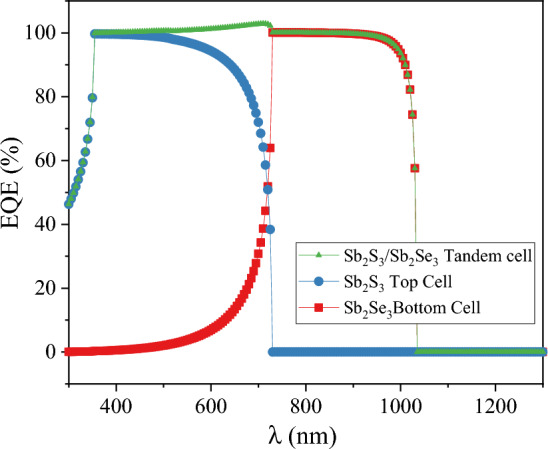
EQE of Sb_2_S_3_ and Sb_2_Se_3_ sub-cells.

Figure 12 illustrates the spectrum of Sb_2_S_3_, Sb_2_Se_3_, and the Sb_2_S_3_/Sb_2_Se_3_ tandem device. Additionally, Fig. 13 illustrates the external quantum efficiency of both sub-cells. As illustrated by the quantum efficiency curve in Fig. 13, photon energy was more effectively taken in at longer wavelengths from the bottom cell with a lower band gap and shorter wavelengths from the top cell with a higher band gap, resulting in the generation of current at a wide range of frequencies. At wavelengths up to 729 nm (hν > 1.7 eV), the upper cell is shown to be dominant in the EQE versus wavelength plots. For longer wavelengths (hν < 1.7 eV) however, the upper cell becomes virtually invisible and absorption from the bottom cell increases, resulting in improved quantum efficiency across a broad range.

## Conclusions

Photovoltaic systems can be improved by using tandem structures. The performance of the Sb_2_S_3_ and Sb_2_Se_3_ sub-cells when operating independently was examined, after which the tandem structure of Sb_2_S_3_/Sb_2_Se_3_ was explored. The tandem structure had a power conversion efficiency that was much higher than the standalone cells. In order to achieve “current matching”—a point at which thesub-cells have the same J_SC_—and reduce current loss due to amismatch of current density, it was necessary to change the thickness of the absorber in tandem cells. For thicker top absorbers, higher thicknesses of bottom absorbers are required for current matching. To minimize material usage we propose that the thickness of top layer choose within 400–430 nm range. This Sb_2_S_3_/Sb_2_Se_3_ tandem solar cell yielded an impressive conversion efficiency of 22.2%, with 420 nm and 1020 nm thick absorber layers for its top and bottom sub-cells respectively. The other photovoltaic parameters are J_SC_ (20.15 mA cm^−2^), V_OC_ (1.02 V), and FF (59.58%). This research on the Sb_2_S_3_/Sb_2_Se_3_ tandem design can lead to the production of high-performance tandem solar cells.

### Supplementary Information

Supplementary Information.

## Data Availability

The data that support the findings of this study are available from the corresponding author upon reasonable request.
