# Supplementary material for: Exploring the potential of standalone and tandem solar cells with Sb2S3 and Sb2Se3 absorbers: a simulation study
Source: Sci Rep. 2023 Dec 19;13:22632. doi: 10.1038/s41598-023-49269-w (PMC10730701; doi:10.1038/s41598-023-49269-w)
Supplement: Supplementary file 1 — Supplementary Information. [file 41598_2023_49269_MOESM1_ESM.docx]

**Appendix A. Supplementary material**

**Exploring the Potential of Standalone and Tandem Solar Cells with Sb_2_S_3_ and Sb_2_Se_3_ Absorbers: A Simulation Study**

Z. Dahmardeh^a^, M. Saadat^a,*^

^a^ Department of Physics, University of Sistan and Baluchestan, Zahedan, Iran

^*^Corresponding author. E-mail address: Saadat@phys.usb.ac.ir (M.Saadat).

Table 1S: Main material parameters used in the numerical simulation [1-3].

| Parameter | Sb_2_S_3_/Sb_2_Se_3_ | HTL | ETL | FTO |
| --- | --- | --- | --- | --- |
| Thickness (nm) | 260 | 90 | 62 | 230 |
| E_g_(eV) | 1.7/ 1.2 | 3.0 | 2.4 | 3.5 |
| χ (eV) | 3.70/ 4.04 | 2.0 | 4.5 | 4.0 |
| ε_r_ | 5/ 18 | 3.0 | 10.0 | 9.0 |
| N_c_ (cm^-3^) | 2.2×10^18^ | 2.5×10^18^ | 2.2×10^18^ | 2.2×10^18^ |
| N_v_ (cm^-3^) | 1.8×10^20^ | 1.8×10^19^ | 1.8×10^19^ | 1.8×10^19^ |
| $v_{\mathrm{th}}^{e}$ [cm/s] | 1×10^7^ | 1.0×10^7^ | 1×10^7^ | 1×10^7^ |
| $v_{\mathrm{th}}^{p}$‏‏[cm/s] | 1×10^7^ | 1.0×10^7^ | 1×10^7^ | 1×10^7^ |
| µ_e_ [cm^2^/Vs] | 0.8/ 15 | 1.0×10^-4^ | 100 | 20 |
| µ_p_[cm^2^/Vs] | 0.2/ 5.1 | 2.0×10^-4^ | 25 | 10 |
|  |  |  |  |  |

Table 2S: Properties of defects at bulk absorber, HTL, ETL, and FTO layers used in simulations [4]

|  | N (cm^-3^) | Energy distribution (eV) | σ_e_ [cm^2^] | σ_h_ [cm^2^] |
| --- | --- | --- | --- | --- |
| Absorber defect 1 | 1.3×10^14^ | 0.50 | 1.0×10^-16^ | 1.0×10^-16^ |
| Absorber defect 1 | 1.0×10^15^ | 0.77 | 1.0×10^-16^ | 1.0×10^-16^ |
| HTL defect | 1.0×10^18^ | single | 1.0×10^-15^ | 1.0×10^-15^ |
| ETL defect | 1.0×10^14^ | single | 1.0×10^-15^ | 1.0×10^-15^ |
| FTO defect | 1.0×10^15^ | single | 1.0×10^-15^ | 1.0×10^-15^ |

Table 3S: Comparison of experimental data [4] and simulation results.

| Cell | V_OC_ (V) | J_SC_ (mA/cm^2^) | FF (%) | Eff (%) |
| --- | --- | --- | --- | --- |
| experimental | 0.655 | 24.1 | 63.5 | 10.0 |
| modeling | 0.653 | 24.1 | 63.5 | 10.0 |

**References:**

[1] M.M. Nicolás-Marín, F. Ayala-Mato, O. Vigil-Galán, M. Courel, Sol. Energy, 224 (2021), pp. 245-252.

[2] M. Saadat, O. Amiri, Sol. Energy, 243 (2022), pp. 163-173.

[3] Z. Dahmardeh, M. Saadat, O. Amiri, Sol. Energy, 262 (2023), p. 111788.

[4] R. Tang, X. Wang, W. Lian, J. Huang, Q. Wei, M. Huang, Y. Yin, C. Jiang, S. Yang, G. Xing, Nat. Energy, 5 (2020), pp. 587-595.
